# Predictors of HIV and Syphilis among Men Who Have Sex with Men in a Chinese Metropolitan City: Comparison of Risks among Students and Non-Students

**DOI:** 10.1371/journal.pone.0037211

**Published:** 2012-05-18

**Authors:** Lan Zhang, Xianbin Ding, Rongrong Lu, Liangui Feng, Xuefeng Li, Yan Xiao, Yuhua Ruan, Sten H. Vermund, Yiming Shao, Han-Zhu Qian

**Affiliations:** 1 Vanderbilt Institute for Global Health, Vanderbilt University School of Medicine, Nashville, Tennessee, United States of America; 2 State Key Laboratory for Infectious Disease Prevention and Control, National Center for AIDS/STD Control and Prevention, Chinese Center for Disease Control, Beijing, China; 3 Chongqing Center for Disease Control and Prevention, Chongqing, China; 4 China Office of the Joint United Nations Program on HIV/AIDS, Beijing, China; Rollins School of Public Health, Emory University, United States of America

## Abstract

**Background:**

Men who have sex with men (MSM) are at a substantial risk of HIV, given rising HIV prevalence in urban China. Adolescent and adult students often take HIV-related risk as part of sexual exploration. We compared the risks of HIV and syphilis infections and risky sexual behaviors between student and non-student among urban MSM.

**Methods:**

Respondent driven sampling approach was used to recruit men who were self-identified as MSM in Chongqing Metropolitan City in southwestern China in 2009. Each participant completed a computer-assisted self-interview which collected demographic and behavioral data, and provided blood specimens for HIV and syphilis testing. Multivariable logistic regression analyses identified predictors for HIV and syphilis infections while comparing student and non-student MSM.

**Results:**

Among 503 MSM participants, 36.4% were students, of whom 84.2% were in college. The adjusted prevalence of HIV infection was 5.5% (95% confidence interval [CI]: 2.1%–10.2%) in students and 20.9% (95% CI: 13.7%–27.5%) in non-students; the adjusted prevalence of syphilis was 4.4% (95% CI: 0.7%–9.0%) in students and 7.9% (95% CI: 3.6%–12.9%) in non-students (*P* = 0.12). Two groups had similar risky sexual behaviors such as number of sexual partners and exchanging sex for money. Multivariate analysis showed that students had lower HIV prevalence than non-students (adjusted odds ratio [AOR]: 0.3; 95% CI: 0.1–0.8) adjusting for age, ethnicity and other variables.

**Conclusion:**

Student MSM have lower HIV and similar syphilis prevalence compared with non-student MSM. However, due to a shorter duration of sexual experience and high prevalence of at-risk sexual behaviors among student MSM, HIV risk might be quite high in students as in non-students.

## Introduction

Men who have sex with men (MSM) represent an increasing proportion of newly reported human immunodeficiency virus (HIV) infections in China [1], [2]. National surveillance data associate 32.5% of total new cases to MSM group in 2009, up from 12.2% in 2007 [1]. HIV prevalence among MSM is particularly high in some large Chinese cities, varying from 0.4–9.9% in Beijing [3], [4], [5] to 8.5–16.8% in Chongqing [6,7 8]. In comparison, it remains relatively low in other cities: 0.9–2.2% in Harbin, Heilongjiang Province [9] and 0.5–3.1% in Jinan, Shandong Province [10], [11]. Sociodemographic and behavioral characteristics may be associated with prevalent HIV infection, e.g. older age, less education, unprotected anal intercourse (UAI), multiple male and/or female sex partners, and co-infection with other sexually transmitted infections (STIs) [12], [13], [14]. Some studies suggest that a significant proportion of MSM in large Chinese cities are college students [15], [16], and that college student MSM may have a lower risk of syphilis compared with non-student MSM [16]. The magnitude of HIV infection in Chinese college student MSM is unknown. We compared HIV risk between student and non-student MSM in a large metropolitan city in southwestern China with high background HIV prevalence among MSM [6], [7], [8].

## Methods

From October-December 2009, MSM were recruited in Chongqing, a city with 34 universities and colleges [17]. Inclusion criteria for participation were men 18 years or older, living and/or working in Chongqing at the time of survey, self-reported oral/anal sex or mutual masturbation with another man during the past 12 months, and willing to provide written informed consent. We used an eligibility screening form to assess whether these criteria were met.

Participants were recruited using respondent driven sampling (RDS) approach [18], [19], [20]. Seven MSM were selected as initial seeds, based on the evaluation of their diversity with respect to demographic characteristics and sub-group memberships, active social networks, and high motivation to recruit peers in their social networks. “Seeds” were each asked to recruit up to three participants, who in turn were asked to recruit a subsequent wave of up to three participants until the target sample size was reached and equilibrium was achieved on variables including age, ethnicity, education, marital status, occupation, duration of living in Chongqing City, having incomes in the last year, and having health insurance. Equilibrium is defined as the estimates of key variables converging around a stable sample composition that does not change during the following waves of recruitment and becomes independent of the initial seeds [18], [20]. All referred participants had to be members of the recruiter’s social network and meet the study eligibility. Study information was printed on the coupons for participants’ reference. Participants were compensated 30 *Yuan* (US$4.50) for their participation in the study and an additional 20 *Yuan* (US$3.00) if they successfully recruited other eligible participants. The study was approved by the institutional review boards of the National Center for AIDS/STD Control and Prevention, Chinese Center for Disease Control and Prevention (China CDC), University of California, San Francisco, and Vanderbilt University.

Each participant completed a computer-assisted self-administered interview (CASI) in a private room. The interview collected information on demographics, sexual behaviors, psychosocial behaviors, HIV testing history, and alcohol and drug use. The CASI system was pilot-tested among MSM volunteers in the real-life survey setting prior to its application to this survey. We followed the protocols of standard screening, confirmatory and quality assurance for HIV-1 and syphilis testing [21]. All serological specimens collected from participants were screened for HIV-1 antibody using enzyme-linked immunosorbent assay (ELISA, Vironostika HIV Uni-Form plus O™, bioMerieux, Holland), and for syphilis using rapid plasma reagin test (RPR, Shanghai Rongsheng Biotech, China). Positive HIV-1 samples were then confirmed by Western Blot for HIV-1 (HIV Blot 2.2 WBTM™, Genelabs Diagnostics, Singapore) and reactive syphilis samples were then confirmed by *Treponema pallidum* particle assay (TPPA, Serodia-TP™, Fujirebio Inc., Tokyo, Japan). All the tests were conducted in certified laboratories at Chongqing CDC. All participants received pre- and post-test HIV counseling. HIV negative individuals were referred to local health providers as needed. HIV positive ones were evaluated by Chongqing CDC staff for eligibility of enrollment into sponsored ART program. Syphilis positive individuals were transferred to the STD clinics in Chongqing CDC or other local hospitals.

Because seven seeds were not recruited by their peers, they were excluded from all analyses [22], [23]. The primary objective of the analyses was to compare HIV prevalence between student and non-student MSM. Therefore, the primary outcome variable was HIV sero-status and the primary predictor variable was occupation, which was categorized as students versus non-students, who were registered as full time students in high school or university/college when the survey was conducted. We considered certain demographic and behavioral variables as potential confounders. The Respondent-Driven Sampling Analysis Tool (RDSAT, V 5.6.0; www.respondentdrivensampling.org) was used to estimate characteristics of MSM and HIV and syphilis prevalence adjusting for personal social network size and recruitment pattern [20]. To compare the crude values of social demographics and sexual behaviors by occupation, Chi-square tests were performed for categorical variables or t-tests with unequal variances for continuous variables. To compare the RDS adjusted values of social demographics and sexual behaviors, univariate logistic regression analyses were conducted, in which RDSAT-generated individual weights for occupation were applied. To assess the relationship between predictor variables and HIV serostatus, univariate logistic regression analyses were performed using RDSAT-generated individual weights for HIV serostatus. Variables significantly associated with both occupation and HIV serostatus at the level of *P*<0.1 in the univariate analyses were included in the multivariable analyses. Multivariable logistic regression model was also constructed using RDSAT-generated individual weights of HIV serostatus. Statistical analyses were carried out using STATA/SE™ V11.2 (StataCorp LP, College Station, Texas, USA).

Recruitment chains, or RDS diagrams, were drawn to show the occupation and HIV serostatus of all network members recruited by seven seeds using NetDraw software (V2.097; www.analytictech.com).

## Results

### Sampling Seeds and their Recruits

**Figure 1:**
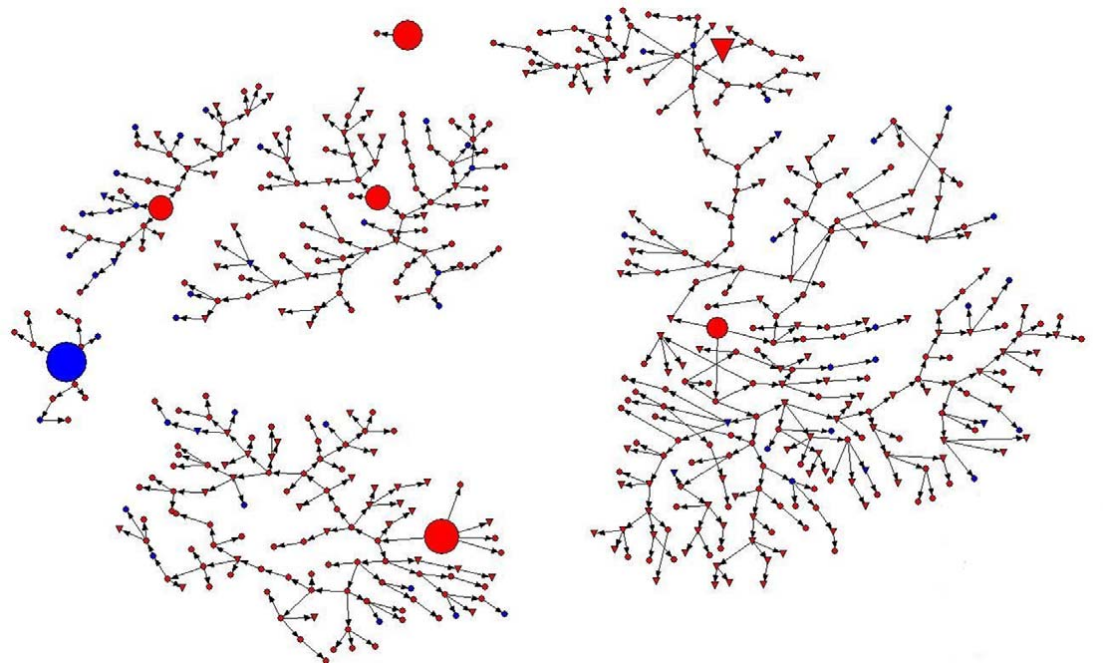
Diagram of respondent driven sampling procedures among MSM in Chongqing City, China: Circles represent non-students while triangles represent students; 7 larger circles or triangles represent the seeds; blue color for HIV-positive and red color for HIV-negative participants.

Study participants were recruited by RDS approach with 7 “seeds” who were also MSM. All 7 seeds were single and Han majority ethnic. They aged 19 to 34 years. Two seeds had high school education, and the rest had college education, including one current college student. One seed was HIV-positive. The final sample reached equilibrium on variables including age, ethnicity, education, marital status, occupation, duration of living in Chongqing City, having incomes in the last year, and having health insurance at the 4^th^ wave of recruitment, and we actually stopped sampling procedures at the 14^th^ wave to have a sufficient sample size for statistical analysis purpose. A total of 506 MSM visited the study clinic for eligibility assessment, 3 were found out ineligible, and 503 participated in the study. The numbers of MSM recruited by 7 seeds were 216, 112, 84, 43, 35, 12, and 1 (Figure 1).

### Socio-demographics of MSM Participants

Of 503 participants, 183 (36.4%) were students, of whom 84.2% were in college. The median age was 23 years (interquartile range [IQR]: 21–26 years), 21 years (IRQ: 19–22 years) among students and 24.5 years (IRQ: 22–28 years) among non-students (*P*<0.001). The median age of having first sex with a man was 20 years (IQR: 18–22 years), 18 years (IQR: 17–20 years) among students and 20 years (IQR: 18–23 years) among non-students (*P*<0.001). More than 90% of participants were of Han ethnicity. More student MSM (86%) received college educations than non-student MSM (61%) (*P*<0.001). Fewer student MSM (0.6%) were married than non-students (15%) (*P*<0.001). Fewer student MSM (15%) were living with male sexual partners (26%) (*P* = 0.008). Students were less likely to have incomes in the past year than non-student MSM (23% versus 88%, *P*<0.001) and less likely to be enrolled in a health insurance plan (32% versus 47%, *P* = 0.008) (Table 1).

**Table 1:** Demographic and behavioral characteristics of 503 men who have sex with men in Chongqing City, China, 2009.

Variable		Unadjusted					Adjusted a			
		Students*N* = 183 (%)	Non-students*N* = 320 (%)	Total*N* = 503 (%)		*P-value*	Students(%, 95% CI)	Non-students(%, 95% CI)	Total (%,95% CI)	*P-value*
**Age (year)**						<0.001				<0.001
** ≤23**		163(89.1)	121(37.8)	284(56.5)			(85.3, 77.4–92.5)	(39.5, 31.7–47.9)	(55.8, 49.0–62.5)	
** >23**		20(10.9)	199(62.2)	219(43.5)			(14.7, 7.5–22.6)	(60.5, 52.1–68.3)	(44.2, 37.5–51.0)	
**Ethnicity**						0.033				0.076
** Han majority**		172(94.0)	313(97.8)	485(96.4)			(90.5, 83.5–96.6)	(96.9, 93.5–99.5)	(94.7, 91.4–97.6)	
** Other minorities**		11(6.0)	7(2.2)	18(3.6)			(9.5, 3.4–16.5)	(3.1, 0.5–6.5)	(5.3, 2.4–8.6)	
**Education**						<0.001				<0.001
** High school or below**		29(15.8)	124(38.8)	153(30.4)			(13.7, 7.6–21.2)	(39.2, 32.3–48.0)	(30.1, 24.8–36.3)	
** College or above**		154(84.2)	196(61.2)	350(69.6)			(86.3, 78.8–92.4)	(60.8, 52.0–67.8)	(69.9, 63.7–75.2)	
**Marital status**						0.001				0.001
** Never married**		182(99.4)	279(87.2)	461(91.7)			(99.4, 98.2–100.0)	(84.8, 78.3–90.7)	(90.0, 85.7–94.0)	
** Ever married** b		1(0.6)	41(12.8)	42(8.3)			(0.6, 0.0–1.8)	(15.2, 9.3–21.7)	(10.0, 6.0–14.3)	
**Currently living with a male sexual partner**						<0.001				0.008
** No**	162(88.5)		239(74.7)	401(79.7)			(85.4, 77.4–92.9)	(73.6, 65.1–79.0)	(77.8, 71.4–82.3)	
** Yes**	21(11.5)		81(25.3)	102(20.3)			(14.6, 7.2–22.6)	(26.4, 21.1–35.0)	(22.2, 17.7–28.6)	
**Duration of living in Chongqing City (year)**						<0.001				<0.001
** ≤21**	166(90.7)		215(67.2)	381(75.7)			(93.5, 89.5–96.7)	(74.5, 67.0–80.3)	(81.4, 76.1–85.5)	
** >21**	17(9.3)		105(32.8)	122(24.3)			(6.5, 3.4–10.6)	(25.5, 19.7–33.1)	(18.6, 14.5–23.9)	
**Having incomes in the past year**						<0.001				<0.001
** No**	147(80.3)		32(10.0)	179(35.6)			(77.0, 68.1–85.7)	(11.8, 6.7–17.4)	(35.1, 28.8–41.2)	
** Yes**	36(20.7)		288(90.0)	324(64.4)			(23.0, 14.3–31.9)	(88.2, 82.6–93.4)	(64.9, 58.8–71.2)	
**Having a health insurance**						<0.001				0.008
** No**	127(69.4)		157(49.1)	284(56.5)			(68.3, 59.1–76.8)	(52.9, 44.4–61.0)	(58.4, 52.0–64.2)	
** Yes**	56(30.6)		163(50.9)	219(43.5)			(31.7, 23.2–40.9)	(47.1, 39.0–55.6)	(41.6, 35.8–48.0)	
**Ever having sex with a woman**						<0.001				0.048
** No**	134(73.2)		170(53.1)	304(60.4)			(66.5, 56.5–77.1)	(54.1, 45.7–62.1)	(58.7, 52.0–64.9)	
** Yes**	49(26.8)		150(46.9)	199(39.6)			(33.5, 22.9–43.6)	(45.9, 37.9–54.3)	(41.3, 35.1–48.0)	
**Duration of having had sex with a man (year)**						<0.001				<0.001
** ≤5**	169(92.3)		221(69.1)	390(77.5)			(92.6, 87.4–97.1)	(75.5, 68.7–82.1)	(81.9, 77.3–86.9)	
** >5**	14(7.7)		99(30.9)	113(22.5)			(7.4, 2.9–12.6)	(24.5, 17.9–31.3)	(18.1, 13.1–22.7)	
**Self reported sexual orientation**						0.727				0.754
** Homosexual**	137(74.9)		244(76.3)	381(75.7)			(71.9, 62.1–81.2)	(72.8, 65.2–79.9)	(72.5, 66.7–78.2)	
** Heterosexual**	46(25.1)		76(23.7)	122(24.3)			(28.1,18.8–37.9)	(27.2, 20.1–34.8)	(27.5, 21.8–33.4)	
**Role in anal sex, if any (n = 480)** c						0.455				0.452
** Largely insertive**	49(26.8)		97(30.3)	146(29.0)			(25.6, 17.9–33.7)	(27.9, 21.1–35.9)	(27.2, 22.0–32.9)	
** Largely receptive**	124(73.2)		210(69.7)	334(71.0)			(74.4, 66.3–82.1)	(72.1, 64.1–78.9)	(72.8, 67.1–78.0)	
**Number of male partners in the past 6 months**						0.965				0.984
** ≤1**	81(44.3)		141(44.1)	222(44.1)			(52.7, 42.9–62.3)	(52.2, 44.8–60.8)	(52.5, 46.7–59.3)	
** >1**	102(55.7)		179(55.9)	281(55.9)			(47.3, 37.7–57.1)	(47.8, 39.2–55.2)	(47.5, 40.7–53.3)	
**Exchanging sex for money in the past 6 months**						0.641				0.706
** No**	175(95.6)		303(94.7)	478(95.0)			(96.1, 92.8–98.8)	(95.2, 91.2–98.3)	(95.5, 92.6–97.8)	
** Yes**	8(4.4)		17(5.3)	25(5.0)			(3.9, 1.2–7.2)	(4.8, 1.7–8.8)	(4.5, 2.2–7.4)	
**Venue for finding the last male sexual partner in the past 6 months (n = 477)** d						0.675				0.940
** Others**	53(30.3)		86(28.5)		139(29.1)		(27.1, 19.0–34.4)	(28.7, 21.7–37.3)	(28.9, 23.5–35.0)	
** Internet**	122(69.7)		216(71.5)		338(70.9)		(72.9, 65.6–81.0)	(71.3, 62.7–78.3)	(71.1, 65.0–76.5)	
**Syphilis infection**						0.307				0.333
** No**	176(96.2)		301(94.2)		477(94.8)		(95.6, 91.0–99.3)	(92.1, 87.1–96.4)	(93.4, 89.9–96.5)	
** Yes**	7(3.8)		19(5.9)		26(5.2)		(4.4, 0.7–9.0)	(7.9, 3.6–12.9)	(6.6, 3.5–10.1)	

a Adjusted values using RDSAT-generated weights for a respondent driven sample.

b Including married, divorced and widowed.

c 23 men reported oral sex or masturbation only.

d 26 men reported no male sexual partner in the past 6 months.

CI: confidence interval.

### HIV Risk Behaviors

Over one in four (28%) of MSM participants perceived themselves as bisexual; 6.5% of students had female sexual partners while 11.6% of non-students did (*P* = 0.200). Non-student MSM were more likely to have had sex with a woman (*P* = 0.048) and to have had sex with men for >5 years than students (*P*<0.001) (Table 1). Of the 480 MSM who had ever had anal sex with their partners, 73% practiced receptive anal intercourse. In the past six months, 48% of MSM had >1 male partner and 5% had exchanged sex for money. The Internet was the means of finding their last sexual partner for 71% of MSM. We saw no differences in self-reported role in anal sex, number of male partners and exchanging sex for money in the past 6 months, and the venue for finding the last male sexual partner in the past 6 months between student and non-student MSM (Table 1). Only 26.2% (123/462) of MSM always used condom with their male partner in the past 6 months and there were no difference between students and non-students (*P* = 0.430). In the past twelve months, 77.5% (314/405) reported drinking alcohol at least monthly, and there was no difference between students and non-students (*P* = 0.977). Drug use was uncommon with 3.0% (12/405) reporting ever using non-injection drugs and 0.5% (2/404) reporting injecting drugs in the past 12 months.

### HIV/syphilis Co-infections and Predictors for HIV Infection

The overall adjusted prevalence of HIV infection was 15.7% (95% confidence interval [CI]: 10.8%–21.8%), syphilis was 6.6% (95% CI: 3.5%–10.2%), and co-infection was 2.0% (95% CI: 0.4%–4.3%). The adjusted prevalence of HIV infection was 5.5% (95% CI: 2.1%–10.2%) in students and 20.9% (95% CI: 13.7%–27.5%) in non-students (*P* = 0.001). Adjusted syphilis rates were 4.4% (95% CI: 0.7%–9.0%) in students and 7.9% (95% CI: 3.6%–12.9%) in non-students (*P* = 0.12). Multivariable logistic regression analysis showed that students had a lower risk of HIV infection than non-students (adjusted odds ratio [AOR]: 0.3; 95% CI: 0.1–0.8); ethnic minorities had a higher risk of HIV infection than Han ethnics (AOR: 8.3; 95% CI: 2.4–29.1); and older age was associated with a higher risk of HIV (AOR: 2.2; 95% CI: 1.0–4.9) (Table 2).

**Table 2:** Factors associated with HIV infection among 503 men who have sex with men in Chongqing City, China, 2009.

Factors	No. of participants	No. of HIV positives (%)	Crude OR (95%CI)	P-value	Adjusted OR (95%CI) a	P-value
**Age (year)**				0.001		0.059
** ≤23**	284	21(7.4)	1.00		1.00	
** >23**	219	37(16.9)	3.7(1.7–8.1)		2.2(1.0–4.9)	
**Ethnicity**				0.05		0.001
** Han majority**	485	53(11.0)	1.00		1.00	
** Other minorities**	18	5(27.8)	4.0(1.0–16.2)		8.3(2.4–29.1)	
**Education**				0.02		0.161
** High school or below**	153	26(16.8)	1.00		1.00	
** College or above**	350	32(9.3)	0.4(0.2–0.9)		0.6(0.2–1.3)	
**Marital status**				0.01		0.281
** Never married**	461	9(21.4)	1.00		1.00	
** Ever married** b	42	49(10.7)	0.3(0.1–0.7)		0.5(0.2–1.7)	
**Currently living with a male sexual partner**				0.36		
** No**	401	45(11.4)	1.00			
** Yes**	102	13(12.3)	0.6(0.2–1.7)			
**Occupation**				0.001		0.012
** Non-student**	381	49(15.3)	1.00		1.00	
** Student**	122	9(4.9)	0.2(0.1–0.5)		0.3(0.1–0.8)	
**Duration of living in Chongqing City (year)**				0.16		
** ≤21**	179	45(12.4)	1.00			
** >21**	324	13(8.9)	0.6(0.3–1.3)			
**Having incomes in the last year**				0.10		
** No**	284	13(7.3)	1.00			
** Yes**	219	45(13.9)	2.1(0.9–5.2)			
**Having a health insurance**				0.66		
** No**	304	34(12.1)	1.00			
** Yes**	199	24(10.9)	0.8(0.4–1.7)			
**Ever having sex with a woman**				0.59		
** No**	390	29(9.7)	1.00			
** Yes**	113	29(14.4)	1.2(0.6–2.7)			
**Duration of practicing homosexual sex with a man (year)**				0.68		
** ≤5**	381	42(10.7)	1.00			
** >5**	122	16(14.5)	1.2(0.5–3.0)			
**Self reported sexual orientation**				0.58		
** Homosexual**	146	45(11.8)	1.00			
** Heterosexual**	334	13(11.0)	1.3(0.5–3.2)			
**Role in anal sex, if any (n = 480)** c				0.55		
** Largely insertive**	222	10(6.9)	1.00			
** Largely receptive**	281	48(14.4)	1.4(0.5–3.7)			
**Number of male partner in the past 6 months**				0.81		
** ≤1**	478	17(7.6)	1.00			
** >1**	25	41(14.7)	1.1(0.5–2.3)			
**Exchanging sex for money in the past 6 months**				0.34		
** No**	365	55(11.6)	1.00			
** Yes**	138	3(11.5)	2.3(0.4–13.0)			
**Venue for finding the last male sexual partner in the past 6 months (n = 477)** d				0.90		
** Non-internet**	139	13(9.2)	1.00			
** Internet (ever)**	338	40(12.0)	1.1(0.4–2.8)			
**Syphilis infection**				0.12		
** No**	477	51(10.7)	1.00			
** Yes**	26	7(26.9)	2.8(0.8–10.3)			

a Adjusted values using RDSAT-generated weights for a respondent driven sample.

b Including married, divorced and widowed.

c 24 men reported oral sex or masturbation only.

d 26 men reported no male sexual partner in the past 6 months.

OR: odds ratio; CI: confidence interval.

## Discussion

Our study found high HIV and syphilis prevalence rates among MSM in Chongqing City. Our finding of high HIV prevalence was consistent with earlier data (range of 10.4–16.8%) in Chongqing MSM [6], [7], [15], and was higher than in most other Chinese cities [5], [10], [14]. It is difficult to explain why HIV prevalence in Chongqing City was higher than in other Chinese cities based on the behavioral data in our study sample. We speculated lower use of condoms during sex and more receptive anal intercourse among MSM, but our study showed that 26.2% of participants consistently used condom with the last partner, which is nearly similar to that in other Chinese cities (33.1%–41.5%) [24]. Receptive anal intercourse was reported by 71% of our respondents, comparable to the higher range of reports from other cities (42.1%–79%) [25]. Chongqing City is near a major drug trafficking route in southwest China, and the neighboring regions are heavily affected by HIV due to injection drug use [26], [27], [28]. Since very few MSM in Chongqing were involved in injection drug use [6], [7], the HIV epidemic might first be introduced to the MSM group through sexual contact with HIV-positive drug users and could thereafter increase due to high transmission efficiency via unprotected anal sex [29]. However, this hypothesis needs to be validated by carefully scrutinizing previous surveillance data and exploring the similarity of HIV genotypes between infected MSM and local drug users.

Another main study finding was that college students constituted a large proportion of MSM in Chongqing and that they had lower prevalence of HIV infection than non-student MSM. Student MSM had no difference in the number of male partners with non-student MSM, indicating they were actively engaged in homosexual activities. Homosexual behaviors have been increasingly common among Chinese college students in recent years. Just 20–30 years ago, sex was a taboo topic in public in China, and sexual encounters were rare among college students [30], [31]. However, since the implementation of the “open door policy” in 1978 and market-oriented economic reforms in the 1980s, Chinese society has undergone dramatic economic development and social changes that greatly transformed social norms and attitudes toward sexuality [32]. Western lifestyles and culture such as openness in talking about sex and tolerance toward pre-marital and homosexual sex have flooded the Chinese mass media [33]. Consequently, the Chinese have become more tolerant toward various sexual practices like homosexuality and pre-marital sex among youths [33], [34], [35]. When students enter college, they are relieved from highly intensive study pressure in high school, escaping their parents’ supervision. Students have ready access to information via the Internet and can connect to social networks, e.g., the gay community. These factors may facilitate a proportion of students’ involvement in homosexual activities with other men, including selling sex to earn money for school or other financial needs or desires [36], [37].

The RDS-adjusted HIV prevalence among student MSM is 5.5%, which is about one quarter of that among non-students (20.9%). However, the actual difference of HIV risk might not be so large for the following reasons: the median age interval between having first sex with a male partner and participating in the study among student MSM is only 3 years (21–18 years), which implies that HIV incidence rate among students might be quite high; in addition, the majority of risk behaviors, such as number of male sexual partners and exchanging sex for money, are not different between students and non-students.

In contrast to the difference of HIV prevalence in two groups, there is no statistically significant difference of syphilis prevalence. The possible reason for these different findings might be: syphilis is a curable disease, and large scale of public health programs, as conducted in Chongqing City in the past several years, could significantly reduce its prevalence in a relatively short time period; in comparison, HIV prevalence is unlikely to decline significantly in a short period even though the intervention programs reduce its incidence rate, as the existing HIV cases may not be removed from this pool of MSM population due to the long survival of this disease.

Study strengths include the novelty of addressing student vs. non-student risk and prevalence. To our knowledge, we are the first to explicitly compare HIV risks in student versus non-student MSM. Existing studies including student MSM sample either did not have HIV prevalence [9], [38], [39] or did not compare HIV infection among students versus non-students [40], [41]. Another strength of this study is its rigorous sampling method. RDS is the best available approach for recruiting hidden populations that has been applied successfully in recruiting injection drug users, female sex workers, and MSM in both developed and developing countries [42], [43], [44], [45]. The RDS method constructed sampling frame during the sampling process that distinguishes itself from traditional non-probability methods such as snowball sampling [19], [20]. The final sample composition is independent of the initial, purposefully selected seeds after 5 to 6 waves of recruitment [18], [20]. The recruitment biases can be assessed by calculation of selection probability and adjusted for in the analysis [20], [22].

Limitations are also noted. First, the RDS method might not recruit a representative sample of the whole MSM population in Chongqing. In theory, the RDS method should generate unbiased estimates [20], [22]. In practice, any violations of the assumptions under which the RDS is applied could end up a failure of an unbiased sample [22], [46]. As we rarely know the accurate characteristics and the size of the MSM population, we could hardly verify whether the sample included all MSM networks [47]. Moreover, both univariable and multivariable analyses in this study were conducted by applying RDSAT-generated weights to dependent variables only (e.g., HIV infection) as much of the comparable literature has done [48], [49], [50]. However, such application is still under development and requires further validation. Second, the students were defined as those who were registered as full time students in high school or college when the survey was conducted; therefore, it is arbitrary categorization of students versus non-students. Recent graduates might have a HIV prevalence rate close to that among current students; this misclassification is likely to result in an underestimation of the difference of HIV prevalence rates between student and non-student MSM. We could not fully exclude the possibility that some students might not have homosexual behaviors but stated they had in order to participate in the study under peer manipulation, which could lead to an underestimation of HIV prevalence. If this happened, the actual HIV prevalence among student MSM would be higher than 5.5%. Finally, interviews on sensitive information such as sexual behaviors are often subject to underreporting (information bias) due to stigma. However, our use of the CASI approach should have reduced this bias.

In conclusion, college students were well represented in MSM population in Chongqing. Student MSM were at high risks of HIV and syphilis acquisition despite of their shorter sexual experience compared with non-students.
